# AUT-MENU Project: A Bicentric Intervention Study to Improve the Meal Acceptance of Subjects with Autism Spectrum Disorder

**DOI:** 10.3390/nu18010165

**Published:** 2026-01-04

**Authors:** Maria Vittoria Conti, Chiara Breda, Ilaria Zambon, Sara Basilico, Stefania Ruggeri, Maria Luisa Scalvedi, Francesca Antonazzi, Hellas Cena

**Affiliations:** 1 Laboratory of Dietetics and Clinical Nutrition, Department of Public Health, Experimental and Forensic Medicine, University of Pavia, 27100 Pavia, Italy; chiara.breda@unipv.it (C.B.); ilaria.zambon01@universitadipavia.it (I.Z.); sara.basilico01@universitadipavia.it (S.B.); hellas.cena@unipv.it (H.C.); 2 Research Centre for Food and Nutrition-CREA, Via Ardeatina, 546, 00178 Roma, Italy; stefania.ruggeri@crea.gov.it (S.R.); marialuisa.scalvedi@crea.gov.it (M.L.S.); francesca.antonazzi@crea.gov.it (F.A.); 3 Clinical Nutrition Unit, Istituti Clinici Scientifici Maugeri Istituto di Ricovero e Cura a Carattere Scientifico, 27100 Pavia, Italy; 4 Italian Institute for Planetary Health, 20156 Milan, Italy

**Keywords:** autism spectrum disorder, collective catering, parent training, nutrition, food selectivity

## Abstract

**Background/Objectives**: Individuals with Autism Spectrum Disorder (ASD) often exhibit low dietary diversity due to Food Selectivity (FS), leading to various forms of malnutrition, such as obesity and/or micronutrient deficiencies. The main objective of the AUT-MENU project is to improve meal acceptance among individuals with ASD. A secondary goal is to evaluate the effectiveness of a nutrition education course for parents of enrolled participants to reduce FS. **Methods**: The study is a bicentric intervention conducted in three care centers (Northern area, Pavia and Milan) and one secondary school (Southern area, Rome), involving individuals with ASD aged 3 to 35 years. The study consists of an observational phase (T0) and an intervention phase (T1). At T0, biographical data, clinical characteristics, and dietary patterns of participants are collected. Based on T0 findings and existing nutritional recommendations for ASD individuals, targeted menus are developed and tested. At T1, the same assessment tools used at T0 will be applied to evaluate intervention effects. Additionally, a nutrition education course for caregivers will be implemented between T0 and T1, with a pre- and post-course knowledge questionnaire to assess its effectiveness. **Results**: This paper reports the results from the care centers in the Northern Area. **Conclusions**: Menu adaptations, developed according to individual preferences and nutritional guidelines, did not significantly modify food consumption but were well tolerated, allowing for an improvement in the nutritional profile of meals without reducing acceptability. These findings support the feasibility of implementing tailored menu strategies in collective catering for individuals with ASD.

## 1. Introduction

Feeding difficulties are a common concern among individuals with autism spectrum disorder (ASD), and caregivers often report mealtime behavioral challenges such as food refusal and restricted food intake. These behaviors are encompassed under the term food selectivity (FS), a condition that lacks a universally accepted definition and up to date is assessed using heterogeneous methodologies such as questionnaires that investigate eating behavior during meals (e.g., Brief Autism Mealtime Behavior Inventory—BAMBI, the Screening Tool of Feeding Problems-STEP) and methods that investigate the quantity and type of different foods consumed, such as food frequency questionnaires (FFQs). As a result, reported prevalence rates of FS vary significantly, ranging from 23% to 69% in children with ASD, compared to 1% to 37% in neurotypical ones [1,2,3,4,5].

FS in individuals with ASD is influenced by several sensory factors, including food texture, color, taste, shape, and temperature, as well as aspects such as dish presentation, packaging, and utensils used [5,6,7,8]. This selective behavior often results in a limited dietary variety, with a preference for foods that are soft or semiliquid in texture, pale in color, mild in taste, and homogenous in appearance [9]. Conversely, there is a marked aversion to bitter, sour, or spicy foods, as well as to dishes with strong odors, bright colors, or high temperatures [9]. These quality characteristics of food often lead to the exclusion of entire food groups, such as certain vegetables or fish [9,10]. Thus, food selectivity increases the risk of nutritional deficiencies, particularly in vitamins A, C, D, zinc, iron, and omega-3 fatty acids, and may also contribute to overweight or obesity, given the high consumption of energy-dense, ultra-processed foods [8]. Beyond nutritional implications, FS also impacts the emotional well-being of caregivers, for whom mealtimes are often described as stressful and frustrating. Nonfunctional caregiver responses, although unintentional, can further exacerbate mealtime anxiety, highlighting the importance of caregiver involvement in managing FS [3,6,11]. Another critical aspect involves the developmental course of FS. While some evidence suggests that eating difficulties may improve with age regardless of interventions, this is not consistent across studies, reinforcing the need for early, targeted interventions to prevent rigid and persistent food-related behaviors into adulthood [6]. Despite increasing attention in the literature, structured interventions in collective catering settings remain scarce, particularly in countries like Italy, where no specific guidelines exist for adapting menus to the sensory and nutritional needs of individuals with ASD. This is especially relevant as many adults with ASD reside in care centers and children regularly consume meals in school settings [12].

To address this gap, the authors previously conducted the pilot study FOOD-AUT [10], which adapted canteen meals for 22 adults with ASD by modifying sensory characteristics such as texture, color, and shape. The study demonstrated promising outcomes, including improved food acceptance and dietary variety. Given the study’s limited scope, there was a need to explore these findings in broader contexts. In fact, the current AUT-MENU project [12] aims to extend this approach to three care facilities and a school that accommodate both children and young adults with ASD. A secondary objective is to assess the feasibility of a structured nutrition and behavioral education course for caregivers, supporting both the dietary and psychosocial dimensions of feeding in this population in the home setting.

## 2. Materials and Methods

### 2.1. Study Design and Setting

The AUT-MENU study is a single-arm pre-post bicentric intervention study (without control) conducted by a multidisciplinary team consisting of researchers from the Laboratory of Dietetics and Clinical Nutrition (LDNC) of the University of Pavia and the Research Centre for Food and Nutrition (CREA) of Rome [12]. The study was carried out in two different regions of Italy. In the northern region, three care centers have been involved: Tiglio Fondazione Onlus in Pavia (TP), Dosso Verde in Milan (DVM), and Dosso Verde in Pavia (DVP). In the central region, the study is currently taking place in one high school in Rome: the “Giuseppe Garibaldi” Agricultural Technical Institute. This paper reports data from the three centers in the Northern region.

The study was registered on ClinicalTrial.gov (registration number: NCT06266377) and approved by the Ethics Committee of the Department of the Nervous System and Behavioral Sciences, on 15 January 2024 (project number 155/23).

**Figure 1:**
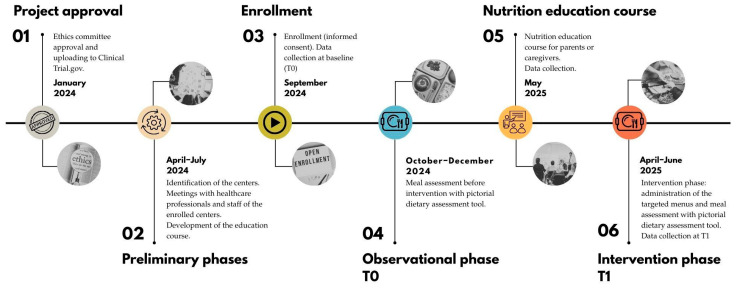
Timeline of the AUT-MENU project.

The study lasted 36 months (November 2022–2025), including a 12-month experimental phase, from September 2024 until September 2025. The timeline of the timeline is shown in Figure 1.

### 2.2. Study Objectives

The primary objective of the AUT-MENU project was to assess meal consumption among individuals with ASD in collective catering settings, both quantitatively (e.g., portion intake) and qualitatively (e.g., sensory characteristics influencing food acceptance, such as texture, color, smell, and shape). Based on these findings and existing dietary guidelines for individuals with ASD [9,10], the project aimed to improve meal acceptance by adapting the proposed meals accordingly.

The secondary objective was to evaluate the feasibility of a structured nutrition and behavioral education program for parents and caregivers of enrolled participants. This intervention aimed to improve parents’ knowledge of FS and meal experience at home, complementing the modifications implemented in the collective catering settings.

### 2.3. Selection Criteria and Enrollment

The study population included children, adolescents, and young adults with a diagnosis of ASD, defined according to the Diagnostic and Statistical Manual of Mental Disorders (DSM-5) [13] diagnostic criteria and encompassing all three severity levels. Participants were recruited from three centers listed above. The eligible age range varied depending on the recruitment site: children and adolescents aged 3 to 17 years were enrolled from the Dosso Verde centers in Milan and Pavia; young adults aged 18 to 35 years were included from Tiglio Fondazione Onlus in Pavia.

Written informed consent was provided by parents or legal guardians of the participants. Exclusion criteria comprised individuals who could not eat independently due to physical or cognitive impairments, those presenting food-related comorbidities such as allergies, intolerances, or medical conditions requiring a specialized diet or artificial nutrition, and participants with other neurological or medical conditions unrelated to ASD (e.g., diabetes, epilepsy) that could influence food consumption or require specific medical management.

In June 2024, the researchers made contact with care centers for individuals with ASD to present the project. Centers that expressed interest were subsequently involved in multi-level presentation sessions addressed both to healthcare professionals (including child neuropsychiatrists, therapists, and educators) and to staff. In September 2024, dedicated meetings were also organized with parents and legal guardians. These sessions had a dual purpose: to inform and train professionals who would act as project facilitators, and to provide parents with detailed information while addressing their questions. During the parental meetings, written informed consent and privacy authorization were collected from parents or legal guardians. Participation in the project was entirely voluntary, and only children, adolescents, or young adults whose parents or legal guardians provided signed consent were enrolled in the study and included in data collection.

### 2.4. Experimental Phase

#### 2.4.1. Meal Assessment and Menu Adaptation

The study was structured into two main phases: an observation phase conducted across all three care centers (T0), followed by an intervention phase carried out in a single center (T1). The observation phase (T0) aimed to assess food consumption from standard menus during lunchtime, focusing on both quantity and sensory characteristics such as shape, color, smell/taste, texture, and temperature. To systematically evaluate these aspects, the pictorial dietary assessment tool (PDAT) was employed [14]. To adequately capture individual variability in dish consumption, a total of 12 PDATs were collected per participant, distributed across three non-consecutive days within a four-week period to ensure robustness and reliability of the dietary assessment. Additional sessions were carried out for those absent to ensure complete data collection. To minimize observer influence and reduce bias, researchers passively observed the participants during lunch without direct interaction. They were responsible for completing the PDATs based on observed consumption patterns. To enhance the accuracy of qualitative data, researchers also tasted and photographed each dish to capture its sensory characteristics comprehensively. At the end of each survey day, the research team convened to align on the organoleptic properties of the meals, ensuring consistency in data interpretation. Data entry was performed daily by two researchers, who decoded the PDATs using a predefined classification system and entered the information into an Excel spreadsheet for further analysis. Based on observed consumption patterns at T0, individual sensory preferences and aversions were inferred (e.g., avoidance of specific colors, temperatures, or textures). These insights were used to inform the adaptation of menus in collaboration with the collective catering service. During the intervention phase (T1), the modified menus were introduced, and the same assessment process used during T0 was repeated.

The objective of this adaptation was to design nutritionally balanced meals that aligned with participants’ sensory preferences, thereby increasing the likelihood of consumption in a real-world catering setting.

#### 2.4.2. Nutrition Education Course for Parents and Caregivers

Between the T0 and T1 phases, an online nutrition education course was offered to subgroups of parents and caregivers of enrolled subjects in the Milan and Pavia centers. The course aimed to support caregivers in managing FS during meals consumed at home. Participation was voluntary. Designed based on healthy eating guidelines and scientific literature on ASD and nutrition [11], the course was led by a multidisciplinary team, including a dietitian, nutrition biologists, speech therapists, and a psychologist. It consisted of seven modules delivered through weekly 1 h sessions, integrating theoretical explanations with practical demonstrations using videos, images, and interactive activities. The topics covered in the modules were as follows:

- Module 1: The role of nutrition on health and macro- and micronutrients.
- Module 2: Food selectivity: associated factors and consequences.
- Module 3: Healthy plate tailored to the needs of individuals with ASD.
- Module 4: Behavioral strategies for the consumption of non-preferred foods.
- Module 5: Sensory strategies for the consumption of non-preferred foods.
- Module 6: Environment and meal presentation.
- Module 7: Mother-child relationship during mealtime.

The course was delivered separately for each participating center. To assess the course’s impact and feasibility, specific evaluation tools (detailed in the following section) were used, along with the recording of attrition rates throughout the program.

### 2.5. Study Data Collection

Data regarding meal consumption were collected by trained researchers and staff working in the participating centers during mealtime. Training consisted of multiple on-site sessions led by the research team, including a detailed explanation of study objectives, standardized tools, and data collection procedures. Prior to study initiation, pilot data collection exercises were conducted at each center. During data collection, at least one researcher from the University team was present in each dining area to supervise procedures, address uncertainties, and ensure protocol adherence. Data regarding both participants and their families were collected through structured phone interviews and then the data were collected into an electronic, web-based Case Report Form (eCRF). Each participant was assigned a unique code to link their data securely while protecting their identity. Details of data collected throughout the study period are presented in the following sections and summarized in Table 1. All the questionnaires used are presented in the study protocol [12].

### 2.6. Primary Outcome

#### 2.6.1. Meal Assessment

The quantity and quality assessment of meals consumed during lunchtime was conducted using the PDAT [14] at both T0 and T1. Rather than relying on the gold standard weighing method, the PDAT offers a less invasive alternative, allowing for uninterrupted mealtime evaluation. Additionally, when compared to the gold standard, it has demonstrated high accuracy and strong correlations with macronutrient and micronutrient intake [14]. The PDAT consists of two sections: a quantitative (Figure S1) and a qualitative component (Figure S2), with the latter developed by researchers based on findings from the FOOD-AUT project [10].

The quantitative section enables the monitoring of each meal component (first course, second course, side dish, accompaniment—bread, breadsticks, crackers—dessert, fruit or yogurt, and beverages) by recording the amount consumed. To facilitate data collection, intake was recorded both numerically (empty plate, 1/4, 1/2, 3/4, and fully consumed) and graphically. The graphic representation employs four circular sectors (representing a plate divided into four sections), which range from white (not consumed) to blue (completely consumed). For beverages, a stylized glass was used, with a gradually increasing water fill indicating empty, one-quarter full, half full, three-quarters full, and full consumption.

The qualitative section evaluated the sensory characteristics of each meal component, detailing its shape, color, smell/taste, texture, and temperature. The development of the qualitative part of the PDAT was finalized with the collaboration of a sensory expert, who helped define the possible alternatives for completing each of the main organoleptic characteristics.

#### 2.6.2. Socio-Demographic Information

At T0, a structured telephone interview was conducted to collect sociodemographic and socioeconomic information. The first section focused on participants with ASD, gathering data on gender, age, ethnicity, clinical characteristics (including pharmacological treatments, therapeutic pathways, anthropometric measurements, weight and height. The second section addressed family-related variables, with particular attention to the primary caregiver, and included information on marital status, age, ethnicity, educational level, as well as strategies adopted for managing food selectivity.

#### 2.6.3. Food Habits and Feeding-Behaviors During Mealtime

At T0, during the same telephone interview, data regarding food habits and meal behaviors of the enrolled participants were also collected. The KIDMED questionnaire (Mediterranean Diet Quality Index for children and adolescents) [15] and the MedQ-Sus questionnaire (New Validated Short Questionnaire for the Evaluation of the Adherence of Mediterranean Diet and Nutrition Sustainability in all adult population groups) [16] were used to assess adherence to the Mediterranean Diet (MD) and to get an overview of the consumption of the main food groups (fruits, vegetables, pasta and cereals, legumes, fish, meat, and dairy products). Adherence to MD diet was rated as low (score ≤ 4), medium (score between 5 and 7), and high (score ≥ 8) for the KIDMED questionnaire and as low (score ≤ 9), medium (score between 9 and 11), and high (score > 11) for the MedQ-Sus questionnaire, while the BAMBI questionnaire (Brief Autism Mealtime Behavior Inventory) [4,17], validated for a population of 3–11 years old, and the STEP questionnaire (Screening Tool for Feeding Problems) [18], validated for a population ≥ 12 years old, were used to evaluate meal-related behavior of subjects with ASD, both at T0 and T1. The presence of feeding difficulties was recorded if the BAMBI questionnaire score was >34. While using STEP, three grades of difficulties were identified: absence or minimal feeding difficulties (score ≤ 7), moderate feeding difficulties (score between 8–11), and severe feeding difficulties (score ≥ 12).

### 2.7. Secondary Outcome

#### 2.7.1. Nutrition Knowledge

Nutrition knowledge was assessed in parents and caregivers participating in the nutrition education course. A questionnaire, designed based on the course topics, consisted of 21 questions (three per module). Researchers administered the questionnaire via telephone interviews before and after the course to evaluate knowledge acquisition. Dichotomous scores were attributed if the answer was right (score = 1) or wrong (score = 0). The maximum score was 21.

#### 2.7.2. Satisfaction with the Course

Satisfaction was evaluated in parents and caregivers attending the course through a 13-question questionnaire. Administered at the end of the program via telephone interviews, it aimed to assess the course’s usefulness, organization, and areas for improvement. The score was based on a Likert scale ranging from 1 (not at all) to 4 (very much) for each question.

### 2.8. Statistical Analysis

In 2024, a total of 78,826 individuals with a diagnosis of Autism Spectrum Disorder (ASD) were registered within the Italian healthcare system (Osservatorio Nazionale Autismo: https://osservatorionazionaleautismo.iss.it/aggiornamento-delle-attivit%C3%A0-2024 accessed on 24 November 2025).

As part of this broader context, we were able to engage and enrol 72 cases at baseline (T0) from three care centres located in the Lombardy region: two in Pavia (*n* = 23 and *n* = 20) and one in Milan (*n* = 23). Notably, the cases attending one centre in Pavia continued the study into T1, with a follow-up sample of 27 participants.

Although the centres differed in their scheduled meal offerings and were attended by individuals with varying age distributions, autism severity levels, and degrees of food selectivity, this diversity allowed for a more nuanced analysis. To account for these differences, the data was analysed across three distinct subgroups, enabling a more tailored interpretation of food-related behaviours.

Given the exploratory nature of the subgroup analyses, no adjustment for multiple testing was applied. These analyses were intended to identify potential patterns rather than to support confirmatory statistical inference. Statistical analyses were performed using IBM SPSS software.

At baseline (T0), a selection of dishes was tested with participants, and at follow-up (T1), the acceptance of newly introduced dishes was assessed.

Food consumption for each menu was calculated as the average across 12 meals, approximating the original discrete variable—defined on a five-point scale—to a continuous variable. The distribution of the mean values of the dishes was found to be non-normal. Therefore, the differences in average consumption among the dishes, as well as within groups defined by demographic and nutritional variables, were assessed using the Kruskal–Wallis test. Unlike ANOVA, this test does not assume normality of the data, making it suitable for ordinal data or continuous data that violate normal distribution assumptions.

## 3. Results

### 3.1. Socio-Demographic Characteristics of the Sample

Subgroup analyses were conducted to explore potential differences across age groups, care settings, and food categories. Given the exploratory nature of these analyses and the number of comparisons performed, the results should be interpreted descriptively rather than inferentially.

Table 2 shows the main socio-demographic characteristics of the sample. The overall sample was predominantly male (77.8%), with females representing 22.2% of the total population. This imbalance was consistent across all care centers. While the DVM and DVP centers included participants with a mean age of 8 (SD, 3.5) and 10.3 (SD, 1.9) years, respectively, the TP center had a higher mean age (25.4 years, SD 4.6), as it hosts adults aged 18 years and older.

Overall, 18.1% of participants were overweight, and 6.8% were affected by obesity, with the highest prevalence observed in the TP center. Conversely, a high prevalence of underweight was recorded in the two centers for minors, DVM and DVP (60.9% and 44.8%, respectively). Regarding ASD severity, no participants presented with level 1 severity; most were classified as level 3 (80.3%).

Concerning food-related behaviors, 40.5% of participants under 12 years of age exhibited feeding difficulties. Among participants aged 12 years and older, 80% showed moderate to severe feeding difficulties (Table 3).

As for adherence to MD, KIDMED scores indicated good overall adherence: among participants under 25 years of age, 51.6% showed medium adherence and 46.8% high adherence. In adults (aged 25 years and older), full adherence to MD was observed (Table 4).

### 3.2. Average Consumption of Each Course at Baseline (T0)

#### 3.2.1. First Course (T0)

Table S1 reports the ranking of average consumption percentage (ACP) for the served first-course dishes, along with the number of meals, variability (SD), and main characteristics of the dishes across the three settings (DVM, DVP, and TP). Overall, ACP was lower in the DVM setting (40.95% ± 46.44) compared with DVP (76.97% ± 38.86) and TP (88.19% ± 28.34). Within DVM, pasta with cheese cream sauce and pizza margherita showed the highest ACP (100% and 81.25%, respectively), while vegetable soup with pasta had the lowest (10.71%). In the DVP setting, most dishes were highly consumed, with ACPs ranging from 58.33% (Roman-style gnocchi) to 85.65% (pasta with pesto sauce). In TP, consumption values were generally high and homogeneous, with most dishes exceeding 80% consumption. Significant associations between ACP and sociodemographic or nutritional indicators were observed. In the DVM setting, pizza margherita was consumed mostly by participants with feeding difficulties (ACP = 94.23%) compared to those without feeding difficulties (ACP = 57.14%, α = 0.03); plain rice was consumed mostly by underweight subjects (ACP = 66.67%) compared to those with normal weight (α = 0.04); vegetable soup with pasta was consumed mostly by subjects aged 16–24 (ACP = 50.00%) compared to those under 11 years (ACP = 4.55%, α = 0.04). In the TP setting, vegetable and legume soup with croutons showed a significantly lower ACP in participants with medium adherence to the KIDMED index (12.5%) compared to low adherence (100.0%) and high adherence (83.3%). Across all settings, dishes characterized by warm temperature, dry texture, and mild smell predominated. Grated cheese was present in most dishes. Multicolored or bi-colored dishes (e.g., pasta with pesto sauce, Roman-style gnocchi) tended to have higher ACPs, whereas mono-colored preparations (e.g., plain rice, vegetable soup) were less consumed, particularly in DVM.

#### 3.2.2. Second Course (T0)

Table S2 presents the ranking of ACP and sensory characteristics of second-course dishes across the three settings (DVM, DVP, and TP). Overall, mean ACP was lowest in DVM (60.62% ± 45.31), intermediate in DVP (70.92% ± 43.21), and highest in TP (90.13% ± 27.79). In the DVM setting, consumption levels were moderate and relatively homogeneous across dishes. The highest ACPs were observed for Roman-style turkey bites (75.00%), followed by fish sticks (64.52%) and codfish balls (58.62%). Plant-based dishes, such as lentil and potato balls and soy balls, showed lower ACPs (53.49% and 45.31%, respectively). All dishes shared similar sensory characteristics, including soft texture, warm temperature, and predominantly mono-colored appearance, with only limited variation in aroma intensity. Statistically significant differences in ACP (α ≤ 0.05) were observed across several demographic and nutritional groups. For lentil and potato balls, ACP reached 100% among overweight and obese participants, compared to 17% in the normal-weight group. Age was also associated with consumption: children under 11 years had an ACP of 42.86%, markedly lower than the 100% recorded in older age classes. For Roman-style turkey bites, foreign nationality was associated with higher ACP (95.5%) compared to Italian nationality (65.2%). For fish sticks, participants with reported feeding difficulties exhibited an ACP of 76.3%, significantly higher than the 45.8% observed in those without such difficulties. In DVP, average ACP values were generally higher. Fish nuggets, fish sticks, chicken cotoletta, and chicken bites had comparable ACPs (approximately 84–87%), followed by braised beef (81.73%) and baked ham (75.00%). Lower ACPs were observed for legume pie (61.00%) and egg omelet (51.92%), while canned tuna had the lowest mean consumption (9.26%). Most second courses were warm and soft, with stronger aromas detected in meat- and fish-based preparations. Significant differences in ACP were identified across dietary quality and demographic groups. For chicken bites, participants with a high KIDMED score had an ACP of 99.1%, compared to 69.8% in the medium KIDMED group. Similarly, for fish sticks, ACP was 100% in the high KIDMED group versus 69.2% in the medium group. For omelets, participants with severe feeding difficulties (STEP) had higher ACP (86.1%) compared to 42.6% in those without difficulties. Age differences were observed, with children under 11 years consuming less (ACP = 39.6%) than those aged 12–16 years (ACP = 71.7%). Nationality also influenced consumption: foreign participants had an ACP of 17.9%, markedly lower than Italians (59.4%). For braised beef, ACP reached 100% in the high KIDMED group compared to 63.5% in the medium group. The TP setting exhibited the highest and most consistent ACPs, with nearly all dishes exceeding 85%. Top-ranked dishes included baked ham (94.40%), beef steaks pizzaiola style (91.67%), and crescenza cheese (91.31%). Even the least consumed dishes, such as braised beef, maintained high acceptance (85.87%). Dishes were mostly mono-colored, with a soft texture and mild smell. A few dishes showed statistically significant differences in ACP. For cooked ham, both low and high KIDMED groups had very high ACPs (100% and 98.9%, respectively), compared to 50.0% in the medium group. Weight status was also associated with consumption: ACP was lower in underweight participants (12.5%) than in normal-weight (84.4%), overweight (94.1%), and obese individuals (100%). For soft cheese (Crescenza), ACPs were high in both low (94.7%) and high KIDMED groups (96.9%), contrasting with 50.0% in the medium group. Finally, for chicken bites, ACP reached 94.2% in the low KIDMED group and 87.5% in the high group, both substantially higher than 25.0% in the medium KIDMED group.

#### 3.2.3. Side Dish (T0)

Table S3 summarizes the ACP and sensory characteristics of side dishes across the three settings (DVM, DVP, and TP). Overall, mean ACP was lowest in DVM (22.79% ± 37.76), higher in DVP (50.77% ± 47.58), and highest in TP (75.00% ± 47.58), showing distinct patterns across the three contexts. In the DVM setting, all side dishes showed low acceptance, with ACPs ranging from 16.1% (cooked white cabbage) to 25.0% (julienne or cooked, rounded carrots). All dishes were mono-colored, with mild or faint smells and mostly soft textures, except for raw preparations, which were described as crunchy. The overall low intake suggests limited acceptance of vegetable-based side dishes in this setting. ACP differed significantly among vegetable dishes (α ≤ 0.05). For sliced carrots, ACP varied by BMI category: underweight participants showed the lowest consumption (6.62%) compared to 32.14% in the normal-weight group and 65.91% in the overweight group. According to the STEP classification, children with no feeding difficulties had an ACP of 17.4%, markedly lower than those with moderate (100%) or severe difficulties (46.4%). For fennel with lemon, ACP was 17.1% in participants without feeding difficulties, significantly lower than 75.0% in those with moderate difficulties. Finally, for cooked white cabbage, ACP was 0% in underweight participants, far below the 62.5% observed in the overweight group. In DVP, consumption increased substantially, with baked potatoes showing the highest ACP (70.8%), followed by broccoli (55.0%) and cooked cabbage (42.4%). Raw or mixed-vegetable dishes, such as white cabbage and rounded carrot salad, had lower ACPs (around 39–41%). Warm, soft-textured, and cooked dishes tended to be preferred, particularly those with more pronounced aromas (e.g., baked potatoes, broccoli). Significant differences in ACP (α ≤ 0.05) were observed for several dishes. For roasted potatoes, ACP was lower among children without feeding difficulties (STEP) compared to those with severe difficulties (63.4% vs. 98.8%). Dietary quality was also associated with consumption: participants with medium adherence to the MD had an ACP of 49.4%, markedly lower than 90% in the high adherence group. Age differences emerged, with children under 11 years consuming less (58.6%) than those aged 12–15 years (87.5%). Nationality played a role as well: foreign participants had an ACP of 48.3%, compared to 75.0% among Italians. For broccoli, ACP was 77.1% in the high adherence group versus 34.6% in the medium group. For cooked white cabbage, ACP reached 74.1% in the high KIDMED group, contrasting with 8.3% in the medium group; children with feeding difficulties consumed more (48.9%) than those without (8.8%), and foreign nationality was associated with lower ACP (16.7%) compared to Italians (49.4%). For sliced carrots, ACP was 72.7% in the high KIDMED group versus 14.2% in the medium group, and foreign participants consumed less (9.37%) than Italians (47.4%). Finally, for white cabbage and tomato salad, ACP was 59.5% in the high KIDMED group versus 16% in the medium group. In the TP setting, side dish consumption was the highest across all settings, with an overall mean ACP of 75%. Baked potatoes ranked first (95.2%), followed by lettuce (88.2%) and raw carrots with corn (81.6%). Even the least-consumed items, such as julienne carrots (67.6%), maintained relatively high acceptance. Both cooked and raw vegetables were well accepted, including mixed or multicolored salads, suggesting broader acceptance of vegetable-based sides in this group. Significant differences in ACP (α ≤ 0.05) were observed for multiple dishes. For broccoli, male participants consumed less (ACP = 59.6%) compared to females (86.6%). For raw carrots with corn, adherence to the MD was associated with ACP: 59.6% in the low adherence group versus 86.6% in the high adherence group. For cooked white cabbage, females showed lower consumption (25%) compared to males (85%). For valerian salad, ACP was highest among participants with low (100%) and high adherence (89.3%) to the MD, both markedly higher than the medium adherence group (25%). Age differences were also evident: participants aged 16–24 years had an ACP of 75.0%, lower than the 100% observed in those over 24 years. For fennel with lemon, ACP was higher in both the low (85%) and high (84.4%) adherence groups, compared to 12.5% in the medium group. Finally, for roasted potatoes, ACP reached 100% in both the low and high adherence groups, while the medium adherence group had 50.0%.

#### 3.2.4. Fruit (T0)

Table S4 summarizes the ACP and sensory characteristics of fruit across the three settings (DVM, DVP, and TP). Overall, mean ACP was lowest in DVM (36.5% ± 45.9), higher in DVP (52.0% ± 47.95), and highest in TP (78.9% ± 39.1), showing distinct patterns across the three contexts. In the DVM setting, extremes in consumption were observed between whole apples with peel (ACP = 85%) and peeled apples (ACP = 13.8%), indicating a strong preference for the unpeeled version. In the DVP setting, bananas had the highest ACP (66.3%), while persimmons had the lowest (36.1%). In TP, bananas were almost completely consumed (ACP = 95%), whereas pears recorded the lowest ACP (78.6%). Analysis of fruit consumption revealed significant differences across groups in the DVM setting. For four fruits—apple with peel, banana without peel, mandarin without peel, and orange without peel—ACP values were significantly higher in children with ASD grade 2 compared to ASD grade 3: 92.31% versus 37.5% for apple, 75% versus 46.79% for banana, 50% versus 9.52% for mandarin, and 45.83% versus 4.17% for orange. These results suggest that children with milder ASD consume these fruits more frequently than those with more severe conditions. Further analysis considering feeding difficulties showed that banana, mandarin, and orange (all without peel) were consumed more by children with feeding challenges than by those without. ACP values were 73.57% versus 31.25% for banana, 39.47% versus 3.57% for mandarin, and 32.5% versus 0% for orange, highlighting the potential influence of texture and ease of consumption on fruit intake among children with feeding difficulties. Regarding body mass index (BMI), fruit consumption varied across weight categories. For bananas, the highest ACPs were observed in overweight or obese children (95% and 87.5%, respectively), while underweight and normal-weight groups showed lower values (54.46% and 2.08%). For mandarin, consumption was concentrated in overweight and obese groups (91.67% and 100%), with no intake recorded among normal-weight participants. In the DVP setting, fruit consumption differed significantly (α ≤ 0.05) between individuals with medium versus high adherence to the MD. For most fruits, ACPs were higher in the high-adherence group. For example, banana intake increased from 51.79% to 83.33% and mandarin from 26.92% to 90.63%. Even fruits with lower consumption in the medium-adherence group, such as persimmons and plums, showed substantial increases (15.4% vs. 55.35% and 11.5% vs. 76%, respectively). For pear without peel, ACP was higher in the overweight group (100%) compared to the underweight group (43.3%), and higher in participants with feeding difficulties (STEP) compared to those without (83.3% vs. 40.5%). Age differences were also observed: participants aged 12–15 years had higher ACPs (71.5%) than children under 11 years (36.9%). ACP was consistently higher for participants of Italian nationality compared to foreign participants across almost all fruit categories. In the TP setting, oranges and mandarins showed very high ACPs in both low- and high-adherence MD groups (orange: 86.6% and 86.5%; mandarin: 84.7% and 94.2%), while the medium-adherence group showed zero consumption for both fruits.

### 3.3. Menu Adaptation

The intervention phase, involving menu adaptation, was conducted in a single care center (DVP). Menu modifications were designed to tailor meal offerings to the specific needs and sensory profiles of participants with ASD, while maintaining nutritional adequacy and complying with the operational constraints of the collective catering service. The adaptation process was guided by three primary sources of information: (i) individual sensory preferences collected at baseline (T0) and findings from the FOOD AUT pilot study [10]; (ii) the Italian dietary guidelines [19]; and (iii) technical and logistical requirements of the collective catering service responsible for meal production. Based on these criteria, menus were modified to enhance both acceptability and nutritional quality. Legumes were incorporated into dry first courses (e.g., pasta with smooth chickpeas and carrot sauce) to increase protein and fiber content while leveraging textures generally well-tolerated according to sensory preference data. Cooked vegetables were also introduced into dry first courses, providing a gentle exposure to vegetables while minimizing the sensory overload associated with raw textures. Vegetables were further added to omelets to create familiar, cohesive textures aligned with participants’ sensory profiles. Given the tendency of many participants to prefer softer and warmer textures, cooked vegetables were systematically favored over raw preparations. Legumes were additionally offered as second-course options in the form of farinata, a chickpea-based dish with uniform texture and mild flavor, consistent with participants’ sensory tolerance. To improve nutritional quality, ultra-processed and pre-fried items were replaced with freshly prepared fish and meat products, either plain or lightly breaded, in line with health guidelines and catering feasibility. Overall, the menu adaptation process reflected a careful balance between sensory suitability, evidence-based nutritional principles, and real-world constraints of large-scale meal preparation, aiming to optimize both acceptability and healthfulness of meals provided to individuals with ASD.

### 3.4. Average Consumption of Each Course After the Menu Adaptation (T1)

Tables S5–S8 report the ranking of the average consumption (%) of dishes and their main sensory characteristics by course (first course, second course, side dish, and fruit) during the intervention phase in a single care center (DVP) (T1).

#### 3.4.1. First Course (T1)

For first courses (*n* = 304 meals), mean consumption was 76.8% (SD 38) (Table S5). The highest ACP values were observed for saffron risotto (85.7%) and pasta with pesto sauce (82.1%), whereas rice with vegetables had the lowest acceptance (52.8%). Most first-course dishes were multicolored, with mild aroma, dry texture, and served warm. Statistically significant differences in ACP (α ≤ 0.05) were observed. For pasta with smooth chickpea and carrot sauce, children with feeding difficulties (BAMBI group) showed higher consumption (ACP = 83.1%) compared to those without difficulties (53.9%). According to the STEP classification, ACP was 60% in the moderate-difficulty group and 100% in the severe-difficulty group. Adherence to the MD also influenced intake: participants with medium adherence had an ACP of 62.8%, markedly lower than 94.4% in the high-adherence group. For pasta with pesto, similar patterns emerged: ACP was 44% in the moderate-difficulty group versus 91% in the severe-difficulty group, and 62.6% in the medium KIDMED group versus 89.6% in the high-adherence group.

#### 3.4.2. Second Course (T1)

For second courses (*n* = 319 meals), mean consumption was 68.7% (SD 44.5) (Table S6). Chicken cutlet (86.1%) and tuna (77.8%) had the highest ACPs, whereas chickpea farinata (44.2%) and zucchini omelet (51.3%) were the least consumed. Meat- and fish-based dishes were generally more appreciated than plant-based options. Most dishes were soft in texture, served warm or at ambient temperature, and monochromatic. Significant differences in ACP (α ≤ 0.05) were observed according to MD adherence. For chicken cutlet, participants with medium adherence showed an ACP of 51.9%, markedly lower than 93.6% in the high-adherence group. Similarly, for chickpea farinata, ACP was 49% in the medium-adherence group compared to 87.5% in the high-adherence group.

#### 3.4.3. Side Dish (T1)

For side dishes (*n* = 325 meals), mean consumption was 45.1% (SD 47.4) (Table S7). The mixed vegetable trio was the most accepted (55.5%), while green salad was the least consumed (36.4%). Cooked vegetables (boiled or baked) were generally preferred, with a soft texture and warm temperature, whereas raw salads were crisp and served at ambient temperature. No significant differences were observed among side dishes overall, but ACP varied significantly according to MD adherence (α ≤ 0.05). For mixed vegetables, participants with medium adherence had an ACP of 33.9%, markedly lower than 72.2% in the high-adherence group. Similar patterns were observed for other dishes: tomato salad with extra virgin olive oil (16.7% vs. 75.9%), boiled green beans (22.7% vs. 68%), and oven-baked diced carrots and zucchini (26.4% vs. 62.1%), with higher ACP consistently associated with greater MD adherence. For tomato salad, children with feeding difficulties (BAMBI) showed higher consumption (ACP = 54.4%) than those without difficulties (12.5%). BMI was also associated with intake: underweight participants had an ACP of 27.1%, markedly lower than 100% in the overweight group. Age differences were evident, with children under 11 years consuming less (32.6%) than those aged 12–15 years (68.2%). For boiled green beans, nationality played a role: foreign participants had an ACP of 9.8%, substantially lower than 52.3% among Italians.

#### 3.4.4. Fruit (T1)

For fruit courses (*n* = 298 meals), mean consumption was 48.2% (SD 48.7) (Table S8). Highest acceptance was recorded for apple (66.7%) and banana (65.4%), whereas watermelon (41.7%) and peach (41.7%) were least consumed. Fruits were served raw, cut into pieces, mostly peeled, and shared similar sensory traits: monochromatic, mild aroma, and ambient temperature. No statistically significant differences were detected among fruit types. Consumption of most fruits was significantly higher among participants with high MD adherence compared to those with medium adherence (α ≤ 0.05). For example, melon and watermelon increased from 25% to 85.7% and from 17.9% to 75.8%, respectively. Similarly, apricot increased from 8.9% to 76.9%, while apple and plum also showed notable increases. These findings suggest that greater adherence to the MD is strongly associated with more frequent intake of a wide variety of fruits.

### 3.5. Consumption Comparison Between T0 and T1 for Each Course at DVP Care Center

Table S9 presents the comparison of average food consumption between two time points (T0 and T1) in the DVP setting. Overall, consumption patterns remained relatively stable, considering that different menu sets were provided in two distinct seasons. Mean consumption of first courses showed a minimal decrease, from 76.97 to 76.81 units, with the number of meals remaining constant (*n* = 304). Second-course consumption declined slightly more, from 70.92 to 68.65 units. Side dish intake decreased from 50.77 to 45.08 units (324 vs. 325 meals), while fruit consumption fell from 51.95 to 48.19 units. These results indicate a modest downward trend in average consumption across meal components over time, particularly for side dishes and fruit.

### 3.6. Parents’ and Caregivers’ Nutritional Knowledge Before and After the Nutrition Education Course

Among all parents and caregivers of the enrolled subjects of the three care centers, the average initial attendance to the nutrition education course was 51.4% (*n* = 38), while the total dropout rate after the course was 34.2% (*n* = 13).

#### 3.6.1. Dosso Verde Pavia

The average score at T0 was 14 out of 21, rising to 17 at T1, corresponding to a +24.6% increase. Out of 7 lectures, the average attendance was 5, making this the center with the highest participation rate. The dropout rate at this center was approximately 68.97%, with 14 of 29 enrolled parents and caregivers not participating in the course and an additional 6 not completing it.

#### 3.6.2. Dosso Verde Milano

The average score at T0 was 16 out of 21, increasing to 18 at T1, with a +6.3% improvement. Average attendance was 3 lectures. The dropout rate at this center was 39.13%. Specifically, of the 23 parents and caregivers enrolled, 5 did not participate in the course, and a further 4 did not complete it.

#### 3.6.3. Tiglio Fondazione Onlus

The average score at T0 was 17 out of 21, increasing to 18 at T1, corresponding to an +8.4% increase. Average attendance was 4 lectures. The dropout rate at this center was the highest (90%), as 15 out of 20 enrolled parents and caregivers did not participate in the course, and an additional 3 did not complete it.

### 3.7. Feasibility of the Nutrition Education Course

Among the parents who participated in the nutrition education course (*n* = 38), *n* = 21 parents responded to the questionnaire related to satisfaction (Table 5) and the majority were mothers (90.5%).

For the questions addressing course organization, perceived engagement during the sessions, satisfaction with the online format, usefulness for improving knowledge about healthy nutrition, and usefulness for increasing understanding of food selectivity and its management, the most frequent response was the maximum score (4). The item regarding the reduction in parental/caregiver stress during mealtimes received an average score of 3, while the item addressing the reduction in stress in children/adolescents with ASD during mealtimes received an average score of 2. Finally, all participating parents and caregivers indicated that they would recommend the course and that they would not prefer in-person teaching.

## 4. Discussion

Food selectivity is a common and persistent challenge in individuals with Autism Spectrum Disorder (ASD), frequently resulting in limited dietary variety and an increased risk of macro- and micronutrient inadequacy. Most interventions addressing food selectivity have focused on psychotherapeutic and behavioral approaches, such as differential reinforcement and alternative behavior training [20,21]. Evidence suggests that early intervention may improve meal acceptance and reduce selective eating behaviors [22,23]. However, nutritional interventions implemented directly within collective catering settings remain limited, particularly across different developmental stages. While previous studies, including the FOOD-AUT pilot study, primarily focused on adults with established eating habits [10], the present study explored the feasibility and acceptability of sensory-informed, nutritionally tailored menu adaptations in children, adolescents, and young adults with ASD. This approach reflects the real-world organization of collective catering services in Italy, where standardized menus are commonly provided to heterogeneous groups within school and adult care settings. Although this heterogeneity complicates the interpretation of aggregated results, it enhances the ecological validity of the study and allows the evaluation of menu adaptations across different developmental stages and living contexts. Despite the integration of evidence-based modifications informed by baseline sensory profiles (T0), prior FOOD-AUT findings, and national dietary guidelines [10,19], no statistically significant changes in overall food consumption were observed following the intervention. Nevertheless, nutritional adequacy remains a key priority, particularly during childhood and adolescence, when appropriate intake supports physical growth and neurodevelopment. Individuals with ASD often consume high amounts of ultra-processed foods (UPFs) [24,25], which may contribute to deficiencies in vitamins (e.g., A, C, folate) and minerals (e.g., iron, zinc) typically provided by fruits and vegetables [26,27,28,29]. In addition, foods such as fish are frequently avoided due to their sensory properties, potentially leading to insufficient intake of calcium, vitamin D, and omega-3 fatty acids [30,31]. To address these nutritional gaps, menu adaptations were implemented in the center where the intervention phase was conducted. Strategies aimed to align sensory preferences with nutritionally relevant foods and included offering cooked rather than raw vegetables, incorporating legumes into familiar preparations, and providing breaded fish products instead of fillets. Importantly, these modifications did not result in reduced food consumption. This finding suggests that foods commonly poorly accepted by individuals with ASD, such as vegetables and legumes, can be introduced in collective catering settings without compromising meal acceptability. The lack of immediate increases in consumption can be interpreted considering the multifactorial nature of eating behaviors in ASD. Food acceptance is influenced not only by sensory characteristics but also by familiarity, established preferences, mealtime routines, and environmental predictability [32]. As a result, repeated exposure and longer adaptation periods may be required before measurable changes in intake occur. In this context, the stability of consumption observed between baseline and follow-up may reflect a transitional adaptation phase rather than a lack of acceptability. Maintaining intake while improving the nutritional quality of meals, therefore, represents a relevant outcome, particularly for foods that are nutritionally valuable yet often under-consumed in ASD populations, such as legumes, cooked vegetables, and lean protein sources [33]. Sample heterogeneity further contributes to the complexity of interpretation. Developmental stage and living context are likely to influence food acceptance and responsiveness to menu adaptations. Younger individuals may exhibit greater sensory sensitivity and food neophobia, whereas adults often show more consolidated eating patterns shaped by long-term routines. Differences between school environments and adult care centers, including meal structure, caregiver support, and social context, may also affect dietary behaviors. For these reasons, subgroup analyses were conducted to explore age- and context-related patterns, and overall results should be interpreted with attention to these differences rather than as uniform effects across the entire sample. The findings also highlight the limitations of menu adaptation as a stand-alone strategy. Future interventions may benefit from integrating sensory-informed menu modifications with complementary approaches, such as repeated exposure protocols, visual supports, and individualized nutritional and behavioral education. In this regard, the observed improvements in parental nutritional knowledge should be interpreted as an intermediate outcome, as behavioral changes in feeding practices may require longer follow-up periods and additional structural support, particularly in families of children with ASD. Given the single-arm pre–post design and the absence of a control group, the present findings must be interpreted with caution. Although menu adaptations were developed based on individual sensory profiles and nutritional guidelines, the observed stability in food consumption cannot be attributed solely to the intervention. Other factors, including seasonal variation, habituation effects, developmental changes, and contextual characteristics of the care settings, may have contributed to the observed patterns. Consequently, no causal inference regarding the effectiveness of the menu adaptations can be drawn. Although this study was conducted within the Italian collective catering system, where organizational and regulatory frameworks may differ from those of other countries, the core principles underlying the intervention—namely sensory-informed menu adaptation, nutritional optimization, and feasibility within institutional food services—are not context-specific. These findings may therefore inform similar strategies in other national and institutional settings, with appropriate adaptation to local food service structures and regulations. Overall, this study provides exploratory evidence that sensory-informed, nutritionally adapted menus are feasible and well tolerated in real-world collective catering settings for individuals with ASD. As the intervention phase was conducted in a single center, the findings are context-specific and should be interpreted primarily as indicators of feasibility and acceptability. Although immediate increases in food consumption were not observed, the results support the potential of a preference-informed, evidence-based approach to improve dietary quality without compromising intake. Further multicentric and controlled studies integrating nutritional, behavioral, and educational strategies are needed to promote healthy eating, inclusivity, and long-term nutritional adequacy in this population.

### Limitations and Strengths of the Study

This study was conducted across three care centres in two Italian cities, providing valuable insights into the food consumption patterns of children, adolescents, and young adults with ASD. However, the sample may not fully capture the diversity of the broader Italian ASD population. Since participants were recruited from care centres, the sample may be biased toward individuals with more pronounced symptoms or higher support needs, potentially underrepresenting those with Level 1 ASD who require minimal assistance. The sample size was inherently limited by the composition of the reference population within the collective catering context, which constrained participant recruitment. Further restrictions arose from the need to standardize meals, leading to the exclusion of individuals requiring special diets (e.g., gluten-free or medically prescribed diets). Additionally, structural constraints imposed by the catering services’ limited menu modifications during the intervention period resulted in full implementation only at the Dosso Verde Centre in Pavia and thereby reducing the effective sample size. Another limitation concerns the uncertain translation of sensory-informed menu recommendations into the home environment, where the majority of evening meals were consumed. This was particularly pronounced among participants whose parents could not attend the online nutritional education program, with digital literacy challenges more evident among older caregivers, especially at the Tiglio Fondazione Onlus centre.

An additional limitation of the present study is the lack of a control or comparison group. While this design choice was driven by ethical, organizational, and real-world constraints within collective catering settings, it limits internal validity and prevents causal conclusions regarding the impact of the menu adaptations. As a result, the findings should be interpreted as descriptive and exploratory, reflecting feasibility and acceptability rather than intervention effectiveness.

Moreover, large numbers of subgroup comparisons performed without correction for multiple testing, which may increase the risk of type I error, are another recognized limitation. Consequently, statistically significant findings from subgroup analyses should be interpreted as exploratory and not as definitive evidence.

Finally, the project’s relatively short duration (one year) precluded the assessment of medium- and long-term health outcomes, such as anthropometric changes or blood markers. Future studies would benefit from longer follow-up periods to evaluate the sustainability of dietary changes and potential health impacts over time.

The wide age range and contextual diversity of the sample represent both a strength and a limitation. While they enhance ecological validity, they limit the comparability of outcomes across subgroups and complicate the interpretation of aggregated results. Future studies with age-stratified designs or context-specific interventions are warranted to better characterize developmental differences in response to menu adaptations.

Despite these limitations, the study presents several notable strengths. To our knowledge, it represents the first nutritional intervention specifically targeting this population, which has historically received limited attention in the research domain. Menus were tailored to the sensory preferences of individuals with ASD, providing a foundation for adaptable meal planning in both collective and domestic settings. The study employed non-invasive methods to collect consumption data, minimizing participant burden and enhancing feasibility. Importantly, the creation of a nutritional education program for parents and caregivers actively involved the home environment, offering a practical, user-friendly tool to support caregivers in optimizing meal management for individuals with ASD. Overall, while methodological constraints limit generalizability, this study provides a pioneering model for sensory-informed, nutritionally optimized interventions that are feasible in real-world settings and highlight the potential for broader implementation in both institutional and home contexts.

## 5. Conclusions

Overall, this study indicates that sensory-informed and nutritionally tailored menu adaptations in collective catering settings for individuals with Autism Spectrum Disorder are feasible and well tolerated. Interpretation of the results should take into account age-related and contextual differences, as food acceptance and dietary behaviors in ASD may vary substantially across developmental stages and care settings. Although the intervention did not result in immediate increases in food consumption, suggesting that the nutritional quality of meals can be enhanced without negatively affecting acceptability. Given the single-arm pre–post design and the absence of a control group, these findings need to be considered context-specific and exploratory and should be interpreted as indicators of feasibility and acceptability rather than evidence of effectiveness. In parallel, the nutrition education course for parents and caregivers proved to be feasible and useful in improving nutritional knowledge and awareness of food selectivity and parental stress management during mealtimes. While causal conclusions cannot be drawn, the combined results support the potential role of integrated, multisystemic approaches that address both institutional and home food environments. Replication of these findings in larger samples, across additional collective catering contexts, and using controlled study designs is warranted to better evaluate the effectiveness of sensory-informed menu adaptations over time. Nevertheless, the present results highlight the importance of developing and implementing tailored nutritional recommendations for individuals with ASD within collective catering services. Policymakers and service providers are encouraged to consider the inclusion of ASD-specific guidelines in public catering systems to promote nutritional accessibility, inclusivity, and long-term dietary quality.

## Figures and Tables

**Table 1:** Summary of data collection tools.

Evaluated Aspects and Tools	Data Regarding	T0	T1
**Meal assessment**			
PDATs	Participants	X	X
**Socio-demographic information**			
CRF	Participants, Parents and Caregivers	X	
**Food habits and MD adherence**			
KIDMED (3–24 years old)	Participants	X	
MedQ-Sus (≥25 years old)			
**Food behaviors**			
BAMBI (3–11 years old)	Participants	X	X
STEP (≥12 years old)			
**Nutrition course**			
Nutrition knowledge questionnaire	Parents and Caregivers	Before and after the course	
Satisfaction Questionnaire		Before and after the course	

Case Report Form (CRF); pictorial dietary assessment tool (PDAT); Mediterranean Diet (MD); Mediterranean Diet Quality Index for children and adolescents’ questionnaire (KIDMED); New Validated Short Questionnaire for the Evaluation of the Adherence of Mediterranean Diet and Nutrition Sustainability in all adult population groups questionnaire (MedQ-Sus); Brief Autism Mealtime Behavior Inventory questionnaire (BAMBI); Screening Tool for Feeding Problems questionnaire (STEP).

**Table 2:** Sociodemographic at baseline (T0).

	DVM *n* = 23	DVP *n* = 29	TP *n* = 20	Total *n* = 72
				
**Age** (years); mean (SD)	8 (3.5)	10.3 (1.9)	25.4 (4.6)	13.8 (6.7)
**Sex**				
Female	3 (13.0%)	8 (27.6%)	5 (25.0%)	16 (22.2%)
Male	20 (87.0%)	21 (72.4%)	15 (75.0%)	56 (77.8%)
**Nationality**				
Italy	18 (78.3%)	24 (82.8%)	20 (100.0%)	62 (86.1%)
Other	5 (21.7%)	5 (17.2%)	0	10 (13.9%)
**BMI classes**				
Underweight	14 (60.9%)	13 (44.8%)	1 (5.0%)	28 (38.9%)
Normal weight	5 (21.7%)	14 (48.3%)	7 (35.0%)	26 (36.1%)
Overweight	3 (13.0%)	2 (6.9%)	8 (40.0%)	13 (18.1%)
Obesity	1 (4.3%)	0 (0.0%)	4 (20.0%)	5 (6.9%)
**ASD severity**				
Grade 2	10 (43.5%)	3 (7.1%)	2 (10.0%)	15 (19.7%)
Grade 3	13 (56.5%)	26 (92.9%)	18 (90.0%)	57 (80.3%)

Categorical variables presented as absolute frequencies (*n*) and percentage (%). Body Mass Index (BMI); autism spectrum disorder (ASD). Dosso Verde Milano (DVM); Dosso Verde Pavia (DVP): Fondazione Tiglio Onlus (TP).

**Table 3:** Feeding difficulties at baseline (T0).

	Age: 3–11 Years *n* = 37
**BAMBI**	
Absence of feeding difficulties	22 (59.5%)
Presence of feeding difficulties	15 (40.5%)
	
	**Age: ≥12 Years** ***n* = 35**
**STEP**	
Absent or minimal feeding difficulties	7 (20.0%)
Moderate feeding difficulties	8 (22.9%)
Severe feeding difficulties	20 (57.1%)

Categorical variables presented as absolute frequencies (*n*) and percentage (%). Bambi questionnaire (Presence of feeding difficulties if score > 34); STEP questionnaire: absent or minimal feeding difficulties (score ≤ 7), Moderate feeding difficulties (score between 8–11), Severe feeding difficulties (score ≥ 12).

**Table 4:** Adherence to the Mediterranean diet at baseline (T0).

	Age: 3–24 *n* = 62
**KIDMED**	
Low	1 (1.6%)
Medium	32 (51.6%)
High	29 (46.8%)
	**Age: 25–35** ***n* = 10**
**MedQ-Sus**	
Low	0 (0.0%)
Medium	0 (0.0%)
High	10 (100.0%)

Categorical variables presented as absolute frequencies (*n*) and percentage (%). KIDMED questionnaire (adherence is rated as low (score ≤ 4), medium (score between 5 and 7), and high (score ≥ 8); MedQ-Sus questionnaire (adherence is rated as low (score ≤ 9), medium (score between 9–11), and high (score > 11).

**Table 5:** Satisfaction Questionnaire (T1).

	Not at All	Slightly	Quite	A Lot
**Course organization**				
1. Do you think the course was well organized?	0 (0%)	1 (4.8%)	8 (38.1%)	12 (57.1%)
2. Did you feel involved during the course lectures?	0 (0%)	3 (14.3%)	8 (38.1%)	10 (47.6%)
3. Did you find the online mode to your liking?	0 (0%)	2 (9.5%)	5 (23.8%)	14 (66.7%)
	**Not at all**	**Slightly**	**Quite**	**A lot**
**Course contents**				
1. In your opinion, was the course content helpful in reducing your stress during mealtime?	2 (9.5%)	7 (33.3%)	8 (38.1%)	4 (19.0%)
2. In your opinion, was the course content helpful in reducing your son/daughter’s stress during mealtime?	3 (14.3%)	8 (38.1%)	7 (33.3%)	3 (14.3%)
3. In your opinion, was the course helpful in improving your knowledge of proper nutrition?	2 (9.5%)	2 (9.5%)	7 (33.3%)	10 (47.6%)
4. In your opinion, was the course helpful in increasing/improving your knowledge about food selectivity and its management?	0 (0%)	3 (14.3%)	7 (33.3%)	11 (52.4%)
5. In your opinion, was the course helpful in expanding your son/daughter’s dietary diversity?	2 (9.5%)	4 (19.0%)	9 (42.9%)	6 (28.6%)
	**No**		**Yes**	
**Other aspects**				
1. Would you prefer the in-person mode?	17 (81.0%)		4 (19.0%)	
2. Would you suggest this course to other parents of children with ASD?	0 (0%)		21 (100%)	

Categorical variables presented as absolute frequencies (*n*) and percentage (%).

## Data Availability

The data that support the findings of this study are available from the corresponding author upon reasonable request, subject to prior authorization from the University of Pavia, the Data Controller in accordance with GDPR. Data sharing is restricted to comply with the regulations of the European Economic Area (EEA). As such, data sharing with entities or individuals located outside the EEA is not foreseen. **Study Registration Number:** ClinicalTrial.Gov NCT06266377.

## Supplementary Materials

The following supporting information can be downloaded at: https://www.mdpi.com/article/10.3390/nu18010165/s1, Figure S1: Dietary Assessment Tool (PDAT), quantitative section; Figure S2: Dietary Assessment Tool (PDAT), qualitative section. Table S1. T0 First course: Ranking of average consumption of the dishes and characteristic aspects per setting; Table S2. T0 Second course: Ranking of average consumption of the dishes and characteristic aspects per setting; Table S3. T0 Side dish: Ranking of average consumption of the dishes and characteristic aspects per setting; Table S4. T0 Fruit: Ranking of average consumption of the dishes and characteristic aspects per setting; Table S5. T1 DVP Ranking of average consumption and characteristic of first course dishes; Table S6. T1 DVP Ranking of average consumption and characteristic of second course dishes; Table S7. T1 DVP Ranking of average consumption and characteristic of side dishes; Table S8. T1 DVP Ranking of average consumption and characteristic of fruit; Table S9. Setting DVP—Comparison of the average consumption between the two measurements by course.

## Abbreviations

The following abbreviations are used in this manuscript:

ACP	Average consumption percentage
ASD	Autism Spectrum Disorder
BAMBI	Brief Autism Mealtime Behavior Inventory questionnaire
CRF	Case Report Form
DVM	Dosso Verde Milano
DVP	Dosso Verde Pavia
FS	Food Selectivity
TP	Fondazione Tiglio Onlus
KIDMED	Mediterranean Diet Quality Index for children and adolescents’ questionnaire
MD	Mediterranean Diet
MedQ-Sus	New Validated Short Questionnaire for the Evaluation of the Adherence of Mediterranean Diet and Nutrition Sustainability questionnaire
PDAT	Pictorial Dietary Assessment Tool
STEP	Screening Tool for Feeding Problems questionnaire
UPFs	Ultra Processed Foods
