# Supplementary material for: AUT-MENU Project: A Bicentric Intervention Study to Improve the Meal Acceptance of Subjects with Autism Spectrum Disorder
Source: Nutrients. 2026 Jan 4;18(1):165. doi: 10.3390/nu18010165 (PMC12787928; doi:10.3390/nu18010165)
Supplement: Supplementary file 1 [file nutrients-18-00165-s001.zip › nutrients-4057315-supplementary.pdf]

nutrients

MDPI

*Type of the Paper (Article)*

# AUT-MENU project: a bicentric intervention study to improve the meal acceptance of subjects with autism spectrum disorder

**Figure S1**. Dietary Assessment Tool (PDAT), quantitative section

Finanziato dall'Unione europea NextGenerationEU · Ministero dell'Università e della Ricerca · Italiadomani PIANO NAZIONALE DI RIPRESA E RESILIENZA · UNIVERSITÀ DI PAVIA · onfoods · AUT menu

## PICTORIAL DIETARY ASSESSMENT TOOL - AUTMENU PROJECT

DATE: ________________ ☐ T0 ☐ T1 OPERATOR: _______________________

SURNAME: ______________________ NAME: __________________________

PLACE:

Dosso Verde Milano
Dosso Verde Pavia
Tiglio Fondazione Onlus

### QUANTITATIVE SECTION

| LUNCH | NULL | ¼ | ½ | ¾ | ALL |
|---|---|---|---|---|---|
| FIRST COURSE | | | | | |
| MAIN COURSE | | | | | |
| SIDE DISH | | | | | |
| BREAD GRISSINI CRACKERS | | | | | |
| FRUIT YOGURT ICECREAM | | | | | |
| BEVERAGES | | | | | |

**Figure S2**. Dietary Assessment Tool (PDAT), qualitative section

# QUALITATIVE SECTION

| LUNCH | GENERAL INFOS | COLOUR | TASTE/SMELL | TEXTURE | TEMPERATURE |
|---|---|---|---|---|---|
| FIRST COURSE | Name: ________________________________<br><br>Pasta type: ______________________<br>❑ Short (e.g. penne, fusilli, rice, barley)<br>❑ Long (e.g. spaghetti, fettuccine)<br><br>Grated cheese?<br>❑ Yes<br>❑ No<br><br>Spices/herbs:<br>❑ Yes<br>❑ No<br>If yes, please specify: _________________ | ❑ White<br>❑ Beige<br>❑ Brown<br>❑ Green<br>❑ Yellow<br>❑ Orange<br>❑ Red<br><br>❑ Monochrome<br>❑ Bicolor<br>❑ Multicolor | ❑ Faint<br>❑ Intense | ❑ Dry<br>❑ Firm/Al dente<br>❑ Soft<br>❑ Sticky/Too dry<br>❑ Untied<br><br>❑ Broth<br>❑ Dense/creamy<br>❑ Liquid<br>❑ Firm/Al dente<br>❑ Soft | ❑ Hot<br>❑ Cold<br>❑ Room temperature |
| MAIN COURSE | Name: __________________________<br>________________________________<br><br>Spices/herbs:<br>❑ Yes<br>❑ No<br>If yes, please specify: _______________<br><br>Breadcrumbs/grating:<br>❑ Yes<br>❑ No | ❑ White<br>❑ Beige<br>❑ Brown<br>❑ Green<br>❑ Yellow<br>❑ Orange<br>❑ Red/Pink<br><br>❑ Monochrome<br>❑ Bicolor<br>❑ Multicolor | ❑ Faint<br>❑ Intense | ❑ Crunchy<br>❑ Firm/Hard<br>❑ Soft<br>❑ Too dry/chewy | ❑ Hot<br>❑ Cold<br>❑ Room temperature |
| SIDE DISH | Name: __________________________<br>❑ Raw<br>❑ Cooked<br><br>Spices/herbs:<br>❑ Yes<br>❑ No<br>If yes, please specify: _______________<br><br>Breadcrumbs/grating:<br>❑ Yes<br>❑ No | ❑ White<br>❑ Beige<br>❑ Brown<br>❑ Green<br>❑ Yellow<br>❑ Orange<br>❑ Red/Pink<br><br>❑ Monochrome<br>❑ Bicolor<br>❑ Multicolor | ❑ Faint<br>❑ Intense | ❑ Crunchy<br>❑ Soft<br>❑ Stringy | ❑ Hot<br>❑ Cold<br>❑ Room temperature |
| BREAD<br>GRISSINI<br>CRACKERS | ❑ Bread<br>❑ Focaccia<br>❑ Crackers<br>❑ Grissini<br><br>Whole grains?<br>❑ Yes<br>❑ No | ❑ White/Beige<br>❑ Brown | ❑ Faint<br>❑ Intense | ❑ Crunchy<br>❑ Soft | ❑ Hot<br>❑ Cold<br>❑ Room temperature |
| FRUIT<br>YOGURT<br>ICECREAM | ❑ Fruit<br>Kind: _____________<br>❑ Whole<br>❑ Cutted<br>❑ Fruit puree<br><br>❑ Raw<br>❑ Cooked<br><br>❑ With peel<br>❑ Whitout peel<br><br>❑ Yogurt<br>❑ Icecream<br>❑ Pudding<br>Flavour: _______________<br>*(Yogurt: fuits, plain, flavored; Pudding: cream/chocolate; Icecream: creams/fruits)* | ❑ White<br>❑ Beige<br>❑ Brown<br>❑ Green<br>❑ Yellow<br>❑ Orange<br>❑ Red/Pink<br>❑ Purple<br><br>❑ Monochrome<br>❑ Bicolor<br>❑ Multicolor | ❑ Faint<br>❑ Intense | ❑ Crunchy<br>❑ Soft<br>❑ Creamy<br>❑ Gelatinous | ❑ Hot<br>❑ Cold<br>❑ Room temperature |
| BEVERAGES | ❑ Water<br>❑ Fruit juice<br><br>How many glasses? __________ | If juice, please specify the flavour:<br>_________________ | ❑ Faint<br>❑ Intense | ❑ Liquid | ❑ Cold<br>❑ Room temperature |

*nutrients* MDPI

**Table S1.** T0 First course: Ranking of average consumption of the dishes and characteristic aspects per setting

| Setting | Number of meals | Average consumption of the dish (%) | SD | Dish | Kind of pasta | Cheese | Color | Texture | Temperature | Smell | Aromas |
|---|---|---|---|---|---|---|---|---|---|---|---|
| DVM | 12 | 100.0[a] | 0.00 | Pasta with cheese cream sauce | *Penne, mezze maniche, sedanini* | Yes | Bicolored | Dry | Warm | Mild | No |
| | 20 | 81.25[b] | 37.06 | Pizza margherita | Pizza | No | Multicolored | Dry | Warm | Mild | No |
| | 16 | 46.88[b] | 43.66 | Plain pasta | *Fusilli, caserecce* | No | Mono colored | Dry | Warm | Mild | No |
| | 17 | 38.24[c] | 48.51 | Plain rice | Rice, barley, *ditalini* | No | Mono colored | Dry | Warm | Mild | No |
| | 19 | 31.58[c] | 41.53 | Pasta with cheese cream sauce | *Penne, mezze maniche, sedanini* | No | Mono colored | Dry | Warm | Mild | No |
| | 16 | 29.69[c] | 45.84 | Ricotta-filled ravioli | *Ravioli* | No | Bicolored | Dry | Warm | Mild | No |
| | 22 | 23.86[c] | 40.44 | *Gnocchi* with tomato sauce | *Gnocchi* | No | Bicolored | Dry | Warm | Mild | No |
| | 16 | 15.63[c] | 34.00 | Roman-style *gnocchi* | Roman-style *gnocchi, polenta* | No | Mono colored | Dry | Warm | Mild | No |
| | 14 | 10.71[c] | 28.95 | Vegetable soup with pasta | *Conchigliette* and *gnocchetti* | No | Mono colored | In broth | Warm | Mild | No |
| | **Total 152** | **40.95** | **46.44** | | | | | | | | |
| DVP | 54 | 85.65 | 31.70 | Pasta with pesto sauce | *Penne, mezze maniche, sedanini* | Yes | Multicolored | Dry | Warm | Mild | No |
| | 27 | 80.56 | 37.55 | Pizza margherita | Pizza | No | Multicolored | Dry | Warm | Mild | No |
| | 73 | 80.48 | 36.14 | Pasta with pesto sauce | *Fusilli, caserecce* | Yes | Multicolored | Dry | Warm | Mild | No |
| | 22 | 78.41 | 41.04 | Pasta with cheese cream sauce | *Penne, mezze maniche, sedanini* | No | Mono colored | Dry | Warm | Mild | No |
| | 30 | 75.83 | 41.77 | Pasta with oil and sage | *Fusilli, caserecce* | Yes | Bicolored | Dry | Warm | Mild | No |
| | 48 | 71.35 | 41.57 | Rice with tomato sauce | Rice, barley, *ditalini* | Yes | Bicolored | Dry | Warm | Mild | No |
| | 24 | 70.83 | 40.15 | Bell pepper cream *risotto* | Rice, barley, *ditalini* | Yes | Bicolored | Dry | Warm | Intense | No |

| | | | | | | | | | | | |
|---|---|---|---|---|---|---|---|---|---|---|---|
| | 14 | 64.29 | 44.63 | Roman-style *gnocchi* | Roman-style *gnocchi, polenta* | No | Mono colored | Dry | Warm | Mild | No |
| | 12 | 58.33 | 51.49 | Roman-style *gnocchi* | Roman-style *gnocchi, polenta* | Yes | Bicolored | Dry | Warm | Mild | No |
| | **Total 304** | **76.97** | **38.86** | | | | | | | | |
| **TP** | 16 | 98.44 | 6.25 | Roman-style *gnocchi* | Roman-style *gnocchi*, polenta | Yes | Bicolored | Dry | Warm | Mild | No |
| | 18 | 94.44 | 23.57 | Pumpkin *risotto* | Rice, barley, *ditalini* | Yes | Bicolored | Dry | Ambient | Mild | No |
| | 15 | 91.67 | 26.16 | Rice with tomato sauce | Rice, barley, *ditalini* | Yes | Bicolored | Dry | Warm | Mild | No |
| | 17 | 89.71 | 26.60 | *Pisarei* and beans | *Conchigliette* and *gnocchetti* | Yes | Multicolored | Dry | Ambient | Mild | No |
| | 16 | 89.06 | 27.34 | Pasta with Parmesan | *Conchigliette* and *gnocchetti* | Yes | Bicolored | Dry | Warm | Mild | No |
| | 15 | 86.67 | 35.19 | Pasta with pesto sauce | *Fusilli, caserecce* | Yes | Multicolored | Dry | Warm | Mild | No |
| | 16 | 82.81 | 37.33 | Vegetable and legume soup with croutons | Vegetable soup | Yes | Bicolored | In broth | Warm | Mild | No |
| | 33 | 80.30 | 31.10 | Rice with potatoes and parsley | Rice, barley, *ditalini* | Yes | Bicolored | In broth | Ambient | Mild | Yes |
| | **Total 146** | **88.19** | **28.34** | | | | | | | | |

*Superscript letters following the mean values of dish consumption indicate significant differences based on Kruskal–Wallis post-hoc pairwise comparisons with α ≤ 0.05. Dosso Verde Milano (DVM); Dosso Verde Pavia (DVP): Fondazione Tiglio Onlus (TP).*

**Table S2.** T0 Second course: Ranking of average consumption of the dishes and characteristic aspects per setting

| Setting | Number of meals | Average consumption of the dish (%) | SD | Dish | Kind of food | Breadcrumbs | Color | Texture | Temperature | Smell | Aromas |
|---|---|---|---|---|---|---|---|---|---|---|---|
| DVM | 34 | 75.00 | 38.44 | Roman-style turkey bites | Chicken, turkey | No | Multicolored | Soft | Warm | Intense | Si |
| | 31 | 64.52 | 41.72 | Fish sticks | Fish | Yes | Mono colored | Soft | Warm | Mild | No |
| | 29 | 58.62 | 46.90 | Codfish balls | Fish | No | Mono colored | Soft | Warm | Mild | No |
| | 43 | 53.49 | 48.36 | Lentils and potatoes balls | Plant-based balls | No | Mono colored | Soft | Warm | Mild | No |
| | 16 | 45.31 | 50.18 | Soy balls | Plant-based balls | Yes | Mono colored | Soft | Warm | Mild | No |
| | **Total 153** | **60.62** | **45.31** | | | | | | | | |
| DVP | 23 | 86.96[a] | 34.44 | Fish nuggets | Fish | Yes | Mono colored | Dry | Warm | Intense | No |
| | 53 | 84.91[a] | 32.65 | Fish sticks | Fish | Yes | Mono colored | Soft | Warm | Intense | No |
| | 28 | 84.82[a] | 35.58 | Chicken *cotoletta* | *Cotoletta* | Yes | Mono colored | Soft | Warm | Mild | No |
| | 58 | 84.48[a] | 34.04 | Chicken bites | Chicken, turkey | No | Mono colored | Soft | Warm | Mild | No |
| | 26 | 81.73[ab] | 38.44 | Braised beef | Meat | No | Mono colored | Soft | Warm | Intense | No |
| | 21 | 75.00[ab] | 41.83 | Baked ham | Meat | No | Mono colored | Soft | Ambient | Mild | No |
| | 25 | 61.00[bc] | 48.99 | Legumes pie | Plant-based balls | No | Bicolored | Soft | Warm | Mild | No |
| | 39 | 51.92[bc] | 46.73 | Egg omelet | Eggs | No | Mono colored | Soft | Warm | Mild | No |
| | 27 | 9.26 [d] | 18.54 | Canned tuna | Tuna | No | Mono colored | Dry | Ambient | Intense | No |
| | **Total 300** | **70.92** | **43.21** | | | | | | | | |
| TP | 58 | 94.40 | 22.48 | Baked ham | Meat | No | Mono colored | Soft | Ambient | Mild | No |
| | 21 | 91.67 | 24.15 | Beef steaks *pizzaiola* style | Meat | No | Bicolored | Hard | Warm | nd | No |

| | | | | | | | | | | |
|---|---|---|---|---|---|---|---|---|---|---|
| 39 | 91.03 | 27.80 | Crescenza cheese | Cheese | No | Mono colored | Soft | Ambient | Mild | No |
| 17 | 89.71 | 29.39 | Spinach omelet | Eggs | No | Mono colored | Soft | Ambient | Mild | No |
| 19 | 88.16 | 31.59 | Swordfish steak | Fish | No | Mono colored | Soft | Ambient | Intense | Yes |
| 43 | 87.79 | 31.52 | Egg omelet | Eggs | No | Mono colored | Soft | Warm | Mild | No |
| 18 | 86.11 | 32.34 | Braised beef | Meat | No | Mono colored | Soft | Warm | Intense | No |
| 23 | 85.87 | 29.99 | Chicken bites | Chicken, turkey | No | Mono colored | Soft | Warm | Mild | No |
| **Total 238** | **90.13** | **27.79** | | | | | | | | |

*Superscript letters following the mean values of dish consumption indicate significant differences based on Kruskal–Wallis post-hoc pairwise comparisons with α ≤ 0.05. Dosso Verde Milano (DVM); Dosso Verde Pavia (DVP): Fondazione Tiglio Onlus (TP).*

**Table S3.** T0 Side dish: Ranking of average consumption of the dishes and characteristic aspects per setting

| Setting | Number of meals | Average consumption of the dish (%) | SD | Dish | Kind of food | Cooked | Color | Texture | Temperature | Smell |
|---|---|---|---|---|---|---|---|---|---|---|
| DVM | 25 | 25.00 | 36.80 | Julienne carrots | Carrots, fennels | Raw | Mono colored | Crunchy | Ambient | Mild |
| | 70 | 25.00 | 39.47 | Cooked rounded carrots | Cooked vegetables | Cooked | Mono colored | Soft | Warm | Mild |
| | 72 | 21.18 | 37.21 | Fennels with lemon vinaigrette | Carrots, fennels | Raw | Mono colored | Crunchy | Ambient | Intense |
| | 14 | 16.07 | 36.17 | Cooked cabbage | Savoy cabbage, fennels | Cooked | Mono colored | Soft | Warm | Intense |
| | **Total 181** | **22.79** | **37.76** | | | | | | | |
| DVP | 95 | 70.79[a] | 44.46 | Baked potatoes | Potatoes | Cooked | Mono colored | Soft | Warm | Mild |
| | 25 | 55.00[ab] | 48.95 | Broccoli | Broccoli, cauliflowers | Cooked | Mono colored | Soft | Warm | Intense |
| | 56 | 42.41[b] | 46.45 | Cooked cabbage | Savoy cabbage, fennels | Cooked | Mono colored | Soft | Warm | Intense |
| | 94 | 40.96[b] | 46.93 | Cooked rounded carrots | Cooked vegetables | Cooked | Mono colored | Soft | Warm | Mild |
| | 54 | 39.35[b] | 44.67 | White cabbage and tomatoes salad | Raw vegetables, mixed salad | Raw | Bicolored | Crunchy | Ambient | Mild |
| | **Total 324** | **50.77** | **47.58** | | | | | | | |
| TP | 21 | 95.24 | 21.82 | Baked potatoes | Potatoes | Cooked | Mono colored | Soft | Warm | Mild |
| | 19 | 88.16 | 26.83 | Lettuce | Carrots, fennels | Raw | Mono colored | Soft | Ambient | Mild |
| | 19 | 81.58 | 37.12 | Raw carrots with corn | Raw vegetables, mixed salad | Raw | Mono colored | Soft | Ambient | Mild |
| | 56 | 80.36 | 38.35 | Broccoli | Broccoli, cauliflowers | Cooked | Mono colored | Soft | Warm | Intense |
| | 21 | 79.76 | 36.76 | Multi-colored salads (lettuce, carrots, tomatoes) | Raw vegetables, mixed salad | Raw | Multicolored | Crunchy | Ambient | Mild |
| | 19 | 78.95 | 41.89 | Boiled carrots and zucchini | Boiled vegetables | Cooked | Bicolored | Soft | Warm | Mild |
| | 20 | 77.50 | 36.18 | Fennels with lemon vinaigrette | Carrots, fennels | Raw | Mono colored | Crunchy | Ambient | Intense |

| | | | | | | | | | | |
|---|---|---|---|---|---|---|---|---|---|---|
| | 20 | 75.00 | 44.43 | Spinach | Cooked vegetables | Cooked | Mono colored | Dry | Warm | Mild |
| | 19 | 72.37 | 44.79 | Cooked cabbage | Savoy cabbage, fennels | Cooked | Mono colored | Soft | Warm | Intense |
| | 17 | 67.65 | 46.57 | Julienne carrots | Carrots, fennels | Raw | Mono colored | Crunchy | Ambient | Mild |
| | **Total 231** | **75.00** | **47.58** | | | | | | | |

*Superscript letters following the mean values of dish consumption indicate significant differences based on Kruskal–Wallis post-hoc pairwise comparisons with α ≤ 0.05. Dosso Verde Milano (DVM); Dosso Verde Pavia (DVP): Fondazione Tiglio Onlus (TP).*

**Table S4.** T0 Fruit: Ranking of average consumption of the dishes and characteristic aspects per setting

| Setting | Number of meals | Average consumption of the dish (%) | SD | Fruit | Cut fruit | Peeled | Cooked | Color | Texture | Temperature | Smell |
|---|---|---|---|---|---|---|---|---|---|---|---|
| DVM | 15 | 85.00$^{a}$ | 35.10 | Apple | Whole | No | Raw | Bicolored | Crunchy | Ambient | Mild |
| | 59 | 56.36$^{b}$ | 47.70 | Banana | Whole | Yes | Raw | Mono colored | Soft | Ambient | Mild |
| | 33 | 24.24$^{c}$ | 41.69 | Mandarin | Cut | Yes | Raw | Mono colored | Soft | Ambient | Intense |
| | 18 | 18.06$^{c}$ | 34.09 | Orange | Cut | Yes | Raw | Mono colored | Soft | Ambient | Intense |
| | 51 | 13.73$^{c}$ | 30.95 | Apple | Cut | Yes | Raw | Mono colored | Crunchy | Ambient | Mild |
| | **Total 176** | **36.51** | **45.90** | | | | | | | | |
| DVP | 52 | 66.35 | 45.88 | Banana | Cut | Yes | Raw | Mono colored | Soft | Ambient | Mild |
| | 41 | 58.54 | 47.96 | Pear | Cut | No | Raw | Bicolored | Soft | Ambient | Mild |
| | 44 | 53.41 | 47.19 | Apple | Cut | Yes | Raw | Mono colored | Crunchy | Ambient | Mild |
| | 21 | 51.19 | 48.40 | Mandarin | Cut | Yes | Raw | Mono colored | Soft | Ambient | Intense |
| | 15 | 50.00 | 44.32 | Apple | Cut | No | Raw | Bicolored | Crunchy | Ambient | Mild |
| | 70 | 49.64 | 48.06 | Pear | Cut | Yes | Raw | Mono colored | Soft | Ambient | Mild |
| | 51 | 43.14 | 50.02 | Plum | Cut | No | Raw | Bicolored | Crunchy | Ambient | Mild |
| | 27 | 36.11 | 47.20 | Persimmon | Cut | Yes | Raw | Mono colored | Gelatinous | Ambient | Mild |
| | **Total 321** | **51.95** | **47.95** | | | | | | | | |
| TP | 21 | 95.24$^{a}$ | 21.82 | Banana | Whole | Yes | Raw | Mono colored | Soft | Ambient | Mild |
| | 64 | 85.16$^{a}$ | 34.99 | Mandarin | Cut | Yes | Raw | Mono colored | Soft | Ambient | Intense |
| | 54 | 83.33$^{a}$ | 34.68 | Orange | Cut | Yes | Raw | Mono colored | Soft | Ambient | Intense |
| | 17 | 82.35$^{a}$ | 36.19 | Mela | Cut | No | Raw | Bicolored | Crunchy | Ambient | Mild |
| | 21 | 80.95$^{a}$ | 37.84 | Mela | Cut | Yes | Raw | Mono colored | Crunchy | Ambient | Mild |

| | | | | | | | | | | | |
|---|---|---|---|---|---|---|---|---|---|---|---|
| | 19 | $80.26^{a}$ | 33.93 | Pera | Cut | Yes | Raw | Mono colored | Soft | Ambient | Mild |
| | 14 | $78.57^{a}$ | 42.58 | Pera | Cut | No | Raw | Bicolored | Soft | Ambient | Mild |
| | 14 | $0.00^{b}$ | 0.0 | Mixed fruits | Purée | Yes | Cooked | Bicolored | Gelatinous | Ambient | Mild |
| | **Total 224** | **78.91** | **39.10** | | | | | | | | |

*Superscript letters following the mean values of dish consumption indicate significant differences based on Kruskal–Wallis post-hoc pairwise comparisons with α ≤ 0.05. Dosso Verde Milano (DVM); Dosso Verde Pavia (DVP): Fondazione Tiglio Onlus (TP).*

**Table S5.** T1 DVP Ranking of average consumption and characteristic of first course dishes

| | Number of meals | Average consumption of the dish (%) | SD | Dish | Kind of pasta | Cheese | Color | Texture | Temperature | Smell | Aromas |
|---|---|---|---|---|---|---|---|---|---|---|---|
| **First course** | 49 | 85.71[a] | 29.32 | Saffron *risotto* | Rice, barley, *ditalini* | Yes | Bicolored | Dry | Warm | Mild | No |
| | 28 | 82.14[a] | 33.92 | Pasta with pesto sauce | *Penne, mezze maniche, sedanini* | Yes | Bicolored | Dry | Warm | Mild | No |
| | 75 | 78.00[a] | 36.75 | Pasta with smooth chickpeas and carrots sauce | *Penne, mezze maniche, sedanini* | Yes | Multicolored | Dry | Warm | Mild | No |
| | 125 | 76.60[a] | 39.24 | Pasta with pesto sauce | *Fusilli, caserecce* | Yes | Multicolored | Dry | Warm | Mild | No |
| | 27 | 52.78[b] | 45.11 | Rice with vegetables | Rice, barley, *ditalini* | Yes | Multicolored | Dry | Warm | Mild | No |
| | **Total 304** | **76.81** | **37.96** | | | | | | | | |

*Superscript letters following the mean values of dish consumption indicate significant differences based on Kruskal–Wallis post-hoc pairwise comparisons with α ≤ 0.05. Dosso Verde Pavia (DVP)*

**Table S6.** T1 DVP Ranking of average consumption and characteristic of second course dishes

| | Number of meals | Average consumption of the dish (%) | SD | Dish | Kind of food | Breadcrumbs | Color | Texture | Temperature | Smell | Aromas |
|---|---|---|---|---|---|---|---|---|---|---|---|
| **Second course** | 52 | 86.06[a] | 33.73 | Chicken cutlet | *Cotoletta* | Yes | Mono colored | Soft | Warm | Mild | No |
| | 54 | 77.78[a] | 40.24 | Tuna | Tuna | No | Mono colored | Soft | Ambient | Intense | No |
| | 58 | 74.14[a] | 42.40 | Chicken scallopini | Chicken, turkey | No | Mono colored | Soft | Warm | Mild | No |
| | 26 | 67.31[a] | 46.78 | Baked fishballs | Fish | No | Mono colored | Soft | Warm | Intense | No |
| | 73 | 58.90[bc] | 47.39 | Chickpeas *farinata* | Plant-based balls | No | Mono colored | Soft | Warm | Mild | No |
| | 56 | 51.34[b] | 47.05 | Zucchini omelet | Eggs | No | Bicolored | Soft | Warm | nd | No |
| | **Total 319** | **68.65** | **44.52** | | | | | | | | |

*Superscript letters following the mean values of dish consumption indicate significant differences based on Kruskal–Wallis post-hoc pairwise comparisons with α ≤ 0.05. Dosso Verde Pavia (DVP)*

**Table S7.** T1 DVP Ranking of average consumption and characteristic of side dishes

| | Number of meals | Average consumption of the dish (%) | SD | Dish | Kind of food | Cooked | Color | Texture | Temperature | Smell | - |
|---|---|---|---|---|---|---|---|---|---|---|---|
| Side dish | 32 | 55.47 | 46.11 | Mixed vegetable trio | Boiled vegetables | Cooked | Multi-colored | Soft | Warm | Mild | - |
| | 55 | 46.82 | 49.31 | Tomato salad with extra-virgin olive oil | Carrots, fennels | Raw | Mono colored | Soft | Ambient | Mild | - |
| | 131 | 44.85 | 48.21 | Boiled green beans | Cooked vegetables | Cooked | Mono colored | Soft | Warm | Mild | - |
| | 72 | 43.75 | 45.34 | Oven-baked diced carrots and zucchini | Cooked vegetables | Cooked | Bicolored | Soft | Warm | Mild | - |
| | 35 | 36.43 | 47.49 | Green salad | Raw vegetables, mixed salad | Raw | Mono colored | Crunchy | Ambient | Mild | - |
| | **Total 325** | **45.08** | **47.41** | | | | | | | | |

*Superscript letters following the mean values of dish consumption indicate significant differences based on Kruskal–Wallis post-hoc pairwise comparisons with α ≤ 0.05. Dosso Verde Pavia (DVP)*

**Table S8.** T1 DVP Ranking of average consumption and characteristic of fruit

| | Number of meals | Average consumption of the dish (%) | SD | Fruit | Cut fruit | Peeled | Cooked | Color | Texture | Temperature | Smell | - |
|---|---|---|---|---|---|---|---|---|---|---|---|---|
| Fruit | 15 | 66.67 | 48.80 | Apple | Cut | No | Raw | Bicolored | Crunchy | Ambient | Mild | - |
| | 26 | 65.38 | 48.52 | Banana | Cut | Yes | Raw | Mono colored | Soft | Ambient | Mild | - |
| | 26 | 57.69 | 50.38 | Apricot | Cut | No | Raw | Mono colored | Soft | Ambient | Mild | - |
| | 61 | 49.18 | 49.36 | Plum | Cut | No | Raw | Bicolored | Soft | Ambient | Mild | - |
| | 27 | 42.59 | 46.93 | Melon | Cut | Yes | Raw | Mono colored | nd | Cold | Intense | - |
| | 89 | 42.25 | 47.67 | Apple | Cut | Yes | Raw | Mono colored | Crunchy | Ambient | Mild | - |
| | 27 | 41.67 | 49.52 | Watermelon | Cut | Yes | Raw | Mono colored | Crunchy | Ambient | Mild | - |
| | 27 | 41.67 | 47.54 | Peach | Cut | No | Raw | Bicolored | Crunchy | Ambient | Mild | - |
| | **Total 298** | **48.19** | **48.65** | | | | | | | | | |

*Superscript letters following the mean values of dish consumption indicate significant differences based on Kruskal–Wallis post-hoc pairwise comparisons with α ≤ 0.05. Dosso Verde Pavia (DVP)*

nutrients

**Table S9.** Setting DVP - Comparison of the average consumption between the two measurements by course

| | T0 | | T1 | |
|---|---|---|---|---|
| | **Number of meals** | **Average consumption of the dish (%)** | **Number of meals** | **Average consumption of the dish (%)** |
| **First course** | 304 | 76.97 | 304 | 76.81 |
| **Second course** | 300 | 70.92 | 319 | 68.65 |
| **Side dish** | 324 | 50.77 | 325 | 45.08 |
| **Fruit** | 321 | 51.95 | 298 | 48.19 |

*Superscript letters following the mean values of dish consumption indicate significant differences based on Kruskal–Wallis post-hoc pairwise comparisons with $\alpha \leq 0.05$. Dosso Verde Pavia (DVP)*
